# Delay Discounting of Monetary and Social Media Rewards: Magnitude and Trait Effects

**DOI:** 10.3389/fpsyg.2022.822505

**Published:** 2022-02-11

**Authors:** Tim Schulz van Endert, Peter N. C. Mohr

**Affiliations:** School of Business and Economics, Freie Universität Berlin, Berlin, Germany

**Keywords:** delay discounting, impulsivity, social media, Instagram, magnitude effect, intertemporal choice, trait effect

## Abstract

Humans discount rewards as a function of the delay to their receipt. This tendency is referred to as delay discounting and has been extensively researched in the last decades. The magnitude effect (i.e., smaller rewards are discounted more steeply than larger rewards) and the trait effect (i.e., delay discounting of one reward type is predictive of delay discounting of other reward types) are two phenomena which have been consistently observed for a variety of reward types. Here, we wanted to investigate if these effects also occur in the context of the novel but widespread reward types of Instagram followers and likes and if delay discounting of these outcomes is related to self-control and Instagram screen time. In a within-subject online experiment, 214 Instagram users chose between smaller, immediate and larger, delayed amounts of hypothetical money, Instagram followers and likes. First, we found that the magnitude effect also applies to Instagram followers and likes. Second, delay discounting of all three reward types was correlated, providing further evidence for a trait influence of delay discounting. Third, no relationships were found between delay discounting and self-control as well as Instagram screen time, respectively. However, a user’s average like count was related to delay discounting of Instagram likes.

## Introduction

Many decisions in life imply a trade-off between the size of rewards and the delay toward attaining them. When dieting, for example, people forgo a smaller, immediate reward (enjoying an unhealthy snack) in favor of a greater benefit (improved health outcomes) in the future. Similarly, saving money implies preferring to wait for a compounded amount instead of spending a smaller amount in the present. These intertemporal trade-offs have been studied thoroughly in the last decades among human and non-human animals (Ainslie, 1975; Green et al., 1981; Mischel et al., 1989; Kirby and Maraković, 1995; Perry et al., 2005). Both have been found to discount rewards as a function of the delay to receiving them; this process is referred to as delay discounting (Mazur, 1987).

In a typical delay discounting experiment, participants are faced with repeated choices between a smaller, immediately available monetary amount (e.g., USD50 today) and a larger, delayed reward (e.g., USD100 in 7 days). The reward amounts and delays are systematically varied and based on the participant’s choices an individual discount rate can be calculated. Various models that seek to explain discounting behavior have emerged, with the hyperbolic decay model (Mazur, 1987) being able to provide the best fit for most empirical data. According to this model, behavior can be mathematically described by the equation *V* = A/(1 + kD), where V is the present value of the future reward, A is the reward amount and D is the delay associated with the reward. The free parameter k represents an individual’s discount rate and is often used as a measure of behavioral impulsivity. The larger the discount rate, the more a future reward is devalued, which characterizes a relatively more impulsive individual.

Several phenomena have been observed in the delay discounting literature, of which two are further investigated in this study. One of the early findings was the magnitude effect (Thaler, 1981; Green et al., 1994; Kirby and Maraković, 1995), which describes the human tendency to discount smaller rewards more steeply than larger rewards, i.e., people behave more impulsively when having to choose between, e.g., USD10 now vs. USD50 in 1 year compared to a setting with, e.g., USD1,000 now vs. USD5,000 in 1 year. This pattern of behavior is at odds with classical economic theory, which posits that intertemporal choices should be consistent if the annual interest rate is the same (Loewenstein and Thaler, 1989). Shefrin and Thaler (1988) initially proposed mental accounting as an explanation for the magnitude effect, according to which small amounts of money are placed into a mental checking account, mainly dedicated to consumption, and large amounts of money are entered into a mental savings account. Waiting for a small amount thus implies forgoing consumption, whereas waiting for a large amount means forgoing interest earnings. If consumption is perceived as more attractive than interest, decision-makers will choose more impulsively for small rewards and less impulsively for large rewards. However, this explanation is made less plausible by the finding that the magnitude effect also occurs with non-monetary rewards [e.g., health (Chapman and Elstein, 1995)], for which the checkings/savings logic is not meaningful. As a more generic alternative, Loewenstein and Prelec (1992) attributed the magnitude effect to the shape of decision-makers’ value function, which is sharply convex for small outcomes but becomes more elastic for large outcomes. According to this account, individuals do not perceive much difference in value between two small outcomes (e.g., 5 units now and 10 units in 6 months), causing them to choose the immediately available option. However, despite having the same ratio, individuals perceive a larger value difference between, e.g., 50 units now and 100 units in 6 months, resulting in choice for the larger, delayed outcome. Thus, decision-makers are sensitive not only to relative but also absolute differences in reward amounts (Prelec and Loewenstein, 1991).

Another phenomenon commonly observed is a trait-like influence on delay discounting, which is demonstrated by the reliability of delay discounting behavior across time, test instruments, context and reward types (Odum et al., 2020). People’s discount rates have been shown to be stable when retested weeks (Beck and Triplett, 2009) or even years (Anokhin et al., 2015) after the initial assessment. Additionally, delay discounting elicited with one type of test is strongly correlated with results obtained with other types of tests (Smith and Hantula, 2008). Lastly, an individual’s discounting behavior in one context or for one type of reward has been found to be predictive of delay discounting in another context and for another reward (Dixon et al., 2003; Johnson et al., 2010; Odum, 2011). For example, an individual behaving impulsively toward food tends to discount entertainment relatively steeply as well (Charlton and Fantino, 2008). While a slight shift in preferences is observed in these studies (suggesting a state influence), an individual’s discount rates remain similar (reflecting a trait influence). The trait perspective on delay discounting is supported by recent evidence of a genetic basis of delay discounting; studies in humans (Anokhin et al., 2011) and rodents (Wilhelm and Mitchell, 2009) have shown that genetic differences can account for a significant portion of inter-individual differences in delay discounting behavior.

The magnitude and the trait effect have been shown for various types of rewards, such as money (Green et al., 1997), food and drinks (Odum et al., 2006; Jimura et al., 2009), entertainment (Friedel et al., 2014), and even abused substances (Giordano et al., 2002). However, the question if these findings extend to the relatively new phenomenon of social media rewards has yet to be addressed. Social media, such as Facebook or Instagram, have become immensely popular since the 2000s and currently have 2.9 billion (Facebook) and 1 billion (Instagram) monthly active users (Facebook Inc, 2021a,b). Instagram is especially prevalent among the segment of 18- to 34-year olds, making up more than 60% of its user base (Statista, 2021). On the platform, users publish pictures and videos, which are saved to the users’ profile page. Other users may choose to like these posts and follow other users’ accounts in order to receive updates about their activities. The number of followers and likes associated with an account have become highly demanded metrics, which even lead to the formation of businesses that sell fake, computer-generated followers and likes in order to artificially boost an account’s popularity. Some popular media have even referred to these metrics as “social currency” (Colcol, 2020). Our main goal in this present study is to investigate if the past findings on the magnitude effect as well as the trait effect of delay discounting can be extended toward the novel rewards of Instagram followers and likes. Thus, the first two hypotheses for this present study are as follows:

H1: Delay discounting of Instagram followers and likes decreases as reward size increases.

H2: Delay discounting of money, Instagram followers and likes are correlated.

To gain a deeper understanding of its underlying processes, delay discounting has been a frequent topic of neuroscientific studies. While the debate about the exact neural regions involved in delay discounting is still ongoing, researchers have found common ground on the central role that self-control processes play in the context of delay discounting (Peters and Büchel, 2011). According to prominent accounts, individuals with greater ability to control thoughts, emotions and behavior can better withstand the temptation of the immediate reward and thus tend to make the less impulsive choice for the larger, delayed reward (McClure et al., 2004; Berns et al., 2007). A recent meta-analysis has indeed shown that self-control is a reliable predictor of delay discounting behavior (Duckworth and Kern, 2011). In this present study, we seek to replicate these findings in the context of social media rewards.

Lastly, recent studies have found an association between screen time, i.e., time spent with a smartphone, laptop or tablet, and delay discounting (Wilmer and Chein, 2016; Schulz van Endert and Mohr, 2020). While the direction of causality is unknown, people who spend more time with digital devices tend to choose more impulsively in delay discounting tasks with monetary rewards. Here, we want to investigate if this relationship also exists when people choose between immediate and delayed Instagram followers and likes. Therefore, our hypotheses 3 and 4 are as follows:

H3: Self-control is negatively correlated with delay discounting of money, Instagram followers and likes.

H4: Screen time is positively correlated with delay discounting of money, Instagram followers and likes.

Our empirical investigation extends previous findings on the magnitude and trait effect of delay discounting, while it also yielded unexpected null findings concerning the relationships with self-control and screen time. We discuss the implications and limitations of this present study and offer possible directions of future research.

## Materials and Methods

### Participants

In total, 218 adult participants (median age 25 years, 55% female) were recruited from the online participant pool Prolific. The sample size was determined to exceed that of related laboratory studies (typically less than 100 participants) while accounting for possibly reduced data quality of an online experiment. After initial screening four participants were excluded from further analyses due to consistency scores below the recommended threshold of 75% (see below), resulting in a final sample size of 214. There were two requirements for participation: first, participants needed to be fluent in English as the experiment used original versions of various scales (see section “Measures”). Second, participants needed to be regular Instagram users, which was defined as using the app at least once per week. Compensation was based on an hourly rate of USD10.50 recommended by Prolific. All participants were informed about the purpose and contents of the study in written form and agreed to the study conditions upon participation.

### Measures

#### Delay Discounting

Participants’ discounting of future rewards was assessed with the 27-item Monetary Choice Questionnaire (MCQ; Kirby et al., 1999). In this questionnaire participants repeatedly choose either a smaller, immediately available or a larger amount of money available in the future. The instrument comprises three groups of nine items based on the magnitude of the larger, delayed reward. The grouping is as follows: small (USD25, USD30, and USD35), medium (USD50, USD55, and USD60) and large (USD75, USD80, and USD85) magnitudes. The smaller, immediate rewards range from USD11 to USD80. The range of delays to the rewards is seven to 186 days, all outcomes being hypothetical in this study. Based on the participant’s choices, an individual discount rate can be calculated under the assumption of hyperbolic discounting. As a simple and atheoretical alternative, the proportion of choices of the larger delayed reward (LDR) can be used as a measure of delay discounting (Myerson et al., 2014), i.e., the lower the proportion, the more the individual devalues future rewards. The MCQ has been used extensively in the literature as its results are comparable to more comprehensive scales (Epstein et al., 2003) as well as to paradigms that use real or potentially real rewards (Lagorio and Madden, 2005). The current data showed high correlations between the LDR proportions and the natural log of the discount parameter k according to Mazur (1987) (all *r*’s < −0.95, all *p*’s < 0.001) for all outcomes, indicating that the LDR measure was accurately assessing participants’ discounting of future rewards.

**FIGURE 1:**
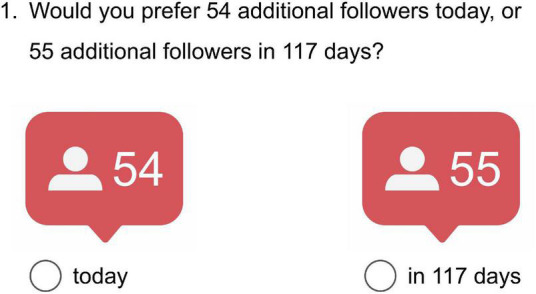
Exemplary choice trial from the delay discounting task for followers.

To assess participants’ delay discounting in an Instagram context, the rewards of the MCQ were simply changed to followers and likes, respectively, while the delays and amounts remained identical. Conveniently, these parameters resemble what a personal Instagram user realistically encounters (fewer than 100 likes, waiting periods of less than 6 months). Additionally, having the same delay ranges and the same number of units of rewards enabled us to use the scoring methodology of the MCQ for both Instagram rewards. To simulate an Instagram setting, in addition to written text, the official Instagram icons showing the respective amounts of followers and likes were used with the corresponding delays displayed below the icons (see Figure 1). To clarify that the offered amounts of followers and likes represented incremental increases rather than the existing balances of followers and likes of participants’ Instagram accounts, the formulations “additional followers” and “additional likes” were used in the choice trials. The responses to the MCQ for all outcomes were scored with Kaplan et al.’s (2014) automated scoring tool. The tool provides the overall LDR proportion (based on all 27 trials) as well as proportions for small, medium and large rewards. In order to identify participants showing insufficient comprehension or lack of effort, the tool also provides consistency scores. Four participants had consistency scores below the recommended threshold of 75% (Kaplan et al., 2016), resulting in their exclusion from the analyses.

#### Self-Control

To measure participants’ trait self-control we used Tangney et al.’s (2004) Brief Self-Control Scale. This widely used scale requires participants to report to what extent they agree with statements such as “I have a hard time breaking bad habits” or “People would say I have iron self-discipline.” The brief 13-item version was employed as it has been shown to be equally reliable and valid as the 36-item version (Tangney et al., 2004). Scores range from 13 to 65, higher scores indicating better ability to control thoughts, emotions and behavior. Good internal consistency was indicated by Cronbach’s α of 0.85.

#### Instagram Preferences

As there may be differences in participants’ goals and attitudes toward Instagram rewards, we included six statements that coarsely elicited participants’ (1) future preference for followers and likes (i.e., how attractive getting followers and likes will be for them personally in the near future) (2) view on the objective future worth of followers and likes (i.e., if they project the worth of followers and likes in the general population to increase or decrease), and (3) motivation to maximize followers and likes (i.e., how important it is for them personally to get as many followers and likes as possible). These statements are based on hypotheses proposed by Odum et al. (2020), which may help explain individual differences in delay discounting behavior. The items were scored on a five-point Likert scale, higher scores representing higher future preference, higher objective future worth and higher motivation, respectively.

#### Additional Variables

Participants also had to estimate their average daily usage duration of Instagram. Next, they stated what year they had joined the platform and whether their profile was private (posts only visible to approved followers) or public (posts visible to anyone). Furthermore, they were asked to indicate the number of followers they had and the average number of likes they typically got on one of their posts. For exploratory purposes, the personality trait of Extraversion was elicited using the 10 Item Personality Measure (Gosling et al., 2003). Lastly, age, gender, education, and discretionary income were elicited as control variables.

### Procedure

All participants underwent the three main experimental conditions of delay discounting of money, Instagram followers and Instagram likes. To control for order effects, Latin Square counterbalancing was employed among these three conditions, i.e., each version of the delay discounting measure occurred only once in any order position. Other measures remained in the same position, resulting in the following order of tasks: (1) MCQ for first outcome (2) Brief Self-Control Scale (3) MCQ for second outcome (4) Ten Item Personality Measure (5) MCQ for third outcome, (6) control variables, and (7) Instagram preferences. The study was conducted in three sessions throughout July 2021 with roughly 70 participants each, one session lasting about 20 min, on average. The study was approved by the German Association for Experimental Economic Research (approval no. x61nvgzI).

### Statistical Analysis

Initially, the distributions of the delay discounting measures were analyzed for normality by means of Shapiro-Wilk tests. Non-parametric analyses were subsequently used for these variables. To investigate differences in delay discounting between different reward sizes (related to hypothesis H1), the Friedman test, as a non-parametric alternative to the repeated-measures ANOVA, was used. As a follow-up analysis, the difference between delay discounting measures for two different reward sizes (e.g., small money vs. medium money) was analyzed with Wilcoxon signed-rank tests. All associations in this study (related to hypotheses H2, H3, and H4 and variables on an ordinal scale) were analyzed by means of Spearman rank correlations with prior log-transformation of highly skewed variables. The difference between correlation coefficients of delay discounting measures for the three reward types was tested with asymptotic *z*-tests. Lastly, for multivariate analyses (related to hypotheses H3 and H4) multiple linear regression was employed.

## Results

### Magnitude Effect of Delay Discounting

**FIGURE 2:**
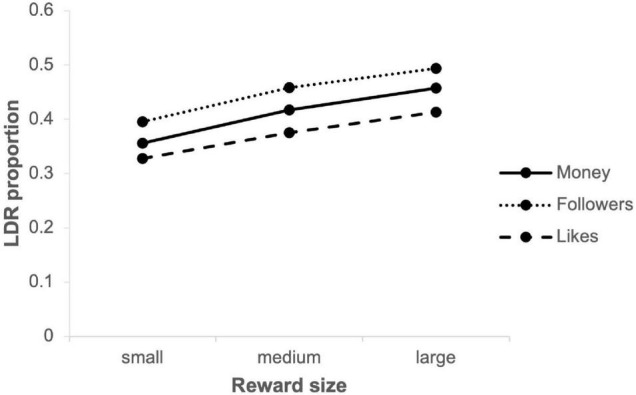
Mean LDR proportions by reward size.

Hypothesis H1 states that delay discounting decreases as reward magnitude increases. Figure 2 shows the mean LDR proportions by reward size for the three different outcomes in this study. The distributions of all proportions appeared approximately normal with a slightly disproportionate number of participants at both extremes; Shapiro-Wilk tests rejected normality (all *p*-values < 0.001). For all outcomes, Friedman tests showed that delay discounting of small, medium and large rewards were statistically different (Money: χ^2^(2) = 142.985, *p* < 0.001; Followers: χ^2^(2) = 102.318, *p* < 0.001; Likes: χ^2^(2) = 95.648, *p* < 0.001). Follow-up Wilcoxon signed-rank tests confirmed that the LDR proportions of medium rewards were higher than of small rewards for all outcomes (all *p*-values < 0.001) and that the LDR proportions of large rewards were higher than of medium rewards for all outcomes (all *p*-values < 0.001), confirming our first hypothesis.

In an attempt to shed light on possible reasons for individual discounting patterns, we investigated the associations between the participants’ stated Instagram preferences with regard to followers and likes and their overall delay discounting of followers and likes (across all reward sizes), respectively. We found the strongest relationship between an individual’s future preference for followers and their overall delay discounting of followers but this relationship was not statistically significant (ρ = −0.11, *p* = 0.126). Furthermore, neither participants’ view on the future worth of followers (ρ = −0.09, *p* = 0.194) nor their motivation to maximize followers (ρ = −0.04, *p* = 0.556) were significantly related to the overall LDR proportion for followers. Looking at the participants’ corresponding attitudes toward likes, neither their future preference for likes (ρ = −0.01, *p* = 0.842) nor their view on the future worth of likes (ρ = 0.00, *p* = 0.990) nor their motivation to maximize likes (ρ = −0.04, *p* = 0.556) were correlated with overall delay discounting of likes.

### Trait Influence of Delay Discounting

Due to the trait-like character of delay discounting, hypothesis H2 states that the LDR proportions for the three outcomes money, followers and likes are correlated. Table 1 shows bivariate Spearman correlations between the main variables in this study. All three LDR measures were significantly intercorrelated, confirming hypothesis 3. The LDR proportions for followers and LDR proportions for likes had the strongest relationship (ρ = 0.60, *p* < 0.001). The correlation between LDR money and LDR likes was ρ = 0.45 with *p* < 0.001, while LDR money and LDR followers showed the weakest association (ρ = 0.35, *p* < 0.001). All three correlation coefficients were significantly different from one another (all *p*-values < 0.05). To further investigate the relationships between delay discounting of these three rewards, we also calculated correlations between the sub-measures (LDR proportions for small, medium and large rewards). As shown in Table 2, within reward types the correlations were all high (all ρ’s > 0.70, all *p*’s < 0.001). Comparing followers with money, small amounts of followers were discounted most similarly as small amounts of money (ρ = 0.41, *p* < 0.001), whereas the weakest correlation was found between the LDR for small amounts of followers and the LDR for large amounts of money (ρ = 0.24, *p* < 0.001). Comparing followers with likes, medium amounts of followers were discounted most similarly as medium amounts of likes (ρ = 0.59, *p* < 0.001), whereas the weakest correlation was found between the LDR for small amounts of followers and the LDR for large amounts of likes (ρ = 0.46, *p* < 0.001). Comparing money with likes, medium amounts of money were discounted most similarly as medium amounts of likes (ρ = 0.48, *p* < 0.001), whereas the weakest correlation was found between the LDR for small amounts of money and the LDR for large amounts of likes (ρ = 0.30, *p* < 0.001).

**TABLE 1:** Correlations between main variables.

Variable	1	2	3	4	5	6	7	8	9	10	11
1. LDR followers	–										
2. LDR money	0.35***	–									
3. LDR likes	0.60***	0.45***	–								
4. Self-control	0.05	0.05	0.05	–							
5. Extraversion	−0.04	0.01	−0.02	0.06	–						
6. Income^a^	−0.13	0.08	−0.06	−0.05	−0.07	–					
7. Instagram screen time^a^	−0.01	−0.02	−0.04	−0.01	−0.02	−0.15*	–				
8. Existing followers^a^	−0.02	−0.01	0.04	0.11	0.22**	−0.01	0.22**	–			
9. Average likes^a^	0.01	−0.00	−0.06	0.11	0.22**	−0.12	0.29***	0.68***	–		
10. Active years	0.05	−0.07	0.05	−0.09	0.11	0.08	0.09	0.31***	0.25***	–	
11. Age^a^	−0.18**	0.09	0.01	−0.07	−0.01	0.30***	−0.14*	−0.22**	−0.45***	0.03	–

*p < 0.05, **p < 0.01, and ***p < 0.001.

*^a^Log transformed.*

*Spearman correlations.*

**TABLE 2:** Correlations between delay discounting sub-measures.

Variable	1	2	3	4	5	6	7	8	9
1. LDR followers large	–								
2. LDR followers medium	0.86***	–							
3. LDR followers small	0.76***	0.81***	–						
4. LDR money large	0.26***	0.27***	0.24***	–					
5. LDR money medium	0.30***	0.35***	0.35***	0.79***	–				
6. LDR money small	0.27***	0.31***	0.41***	0.73***	0.80***	–			
7. LDR likes large	0.52***	0.53***	0.46***	0.35***	0.41***	0.30***	–		
8. LDR likes medium	0.58***	0.59***	0.53***	0.42***	0.48***	0.39***	0.84***	–	
9. LDR likes small	0.50***	0.53***	0.49***	0.37***	0.44***	0.39***	0.77***	0.80***	–

***p < 0.001.

*Spearman correlations.*

### Correlates of Delay Discounting

Hypotheses H3 and H4 state that self-control is negatively correlated and that screen time is positively correlated with delay discounting of money, followers and likes. Therefore, the associations of both self-control and Instagram screen time with the three measures of delay discounting, respectively, were analyzed. As shown in Table 1, neither self-control nor Instagram screen time were correlated with any of the three LDR proportions. Notably, self-control was not correlated with any of the main variables in this study. Screen time was positively related to participants’ amount of existing followers (ρ = 0.22, *p* < 0.01) and to the average number of likes participants receive for a post (ρ = 0.29, *p* < 0.001). To analyze associations between the main variables simultaneously, we performed three multiple regression analyses with the LDR proportions for the three reward types as dependent variables and self-control, extraversion and demographic as well as Instagram-related measures as independent variables. All assumptions for multiple linear regression were met. The results of these analyses confirmed that neither self-control nor Instagram screen time could predict delay discounting of money, followers or likes. However, the average number of likes a person typically receives for an Instagram post significantly predicted the LDR proportion of likes (β = −0.26, *p* = 0.016) when accounting for psychological and demographic variables, as displayed in Table 3. The overall model yielded an *R*^2^ of 0.05, F-statistic of 0.77 and *p*-value of 0.70.

**TABLE 3:** Multiple regression analysis of LDR likes.

Term	B	SE B	95% CI		β	*t*	*p*
			LL	UL			
Intercept	0.47	0.27	−0.07	1.00	0.00	1.72	0.087
Self-control	0.00	0.00	0.00	0.01	0.11	1.46	0.146
Extraversion	0.00	0.01	−0.02	0.02	−0.01	−0.17	0.863
Instagram screen time^a^	0.00	0.01	−0.03	0.03	−0.01	−0.12	0.904
Existing followers^a^	0.02	0.01	−0.01	0.05	0.16	1.61	0.110
Average likes^a^	−0.04	0.02	−0.07	−0.01	−0.26	−2.43	0.016
Profile (private)	−0.01	0.02	−0.04	0.02	−0.05	−0.70	0.487
Active years	0.01	0.01	−0.01	0.02	0.09	1.11	0.267
Age^a^	−0.04	0.07	−0.18	0.10	−0.05	−0.59	0.558
Gender (Female)	0.00	0.02	−0.03	0.03	0.02	0.27	0.786
Education (Bachelor)	0.02	0.03	−0.05	0.09	0.05	0.53	0.597
Education (High school)	−0.01	0.04	−0.08	0.06	−0.04	−0.39	0.695
Education (Master/Diploma)	0.03	0.04	−0.05	0.11	0.06	0.78	0.434
Education (Other)	−0.02	0.06	−0.14	0.10	−0.02	−0.33	0.745
Income^a^	−0.01	0.01	−0.04	0.01	−0.08	−1.12	0.265

*^a^Log transformed.*

*Effect coding was applied for categorical variables.*

## Discussion

The primary goal of this study was to investigate if two well-known phenomena of delay discounting, namely the magnitude effect and the trait effect, also occur in the context of the novel reward types of Instagram followers and likes. Looking initially at the effect of varying reward magnitudes, the present data showed that small rewards were discounted more steeply than medium rewards and that medium rewards were discounted more steeply than large rewards. This magnitude effect occurred for all three outcomes in this study, i.e., money, Instagram followers and likes, which confirmed hypothesis H1. Thus, with the MCQ modified for Instagram rewards, we were able to replicate past findings on the magnitude effect with monetary (e.g., Thaler, 1981; Green et al., 1994; Kirby and Maraković, 1995) and non-monetary (e.g., Baker et al., 2003; Estle et al., 2007; Lawyer et al., 2010) rewards. A prevalent account holds that the magnitude effect is due to the shape of the decision-maker’s value function being convex for small gains and straightening out for large gains (Loewenstein and Prelec, 1992). This property implies that when comparing two equivalent ratios (e.g., USD5/USD1 and USD500/USD100), the ratio involving larger gains is perceived as larger, resulting in less impulsive choices. The present results suggest that Instagram followers and likes, being new, non-consumable rewards from the digital sphere, are subject to a value function with a similar curvature as other well-researched outcomes, such as money, food and drinks, entertainment or addictive substances. In an Instagram user context the magnitude effect implies that people seem to be more impulsive when the number of additional followers and likes they receive is relatively lower, such as when they have posted less popular content. In contrast, when a photograph or video post is being received more positively users seem to be more willing to wait for any additional followers and likes. An interesting question for future research could be if this shift in impulsivity spills over onto behavior outside the platform, i.e., do people choose more impulsively (e.g., at work, while shopping) when they have posted content on social media which is receiving relatively less appreciation? Such an effect, if observed, would then have to be disentangled from any mood changes induced by relatively less positive social feedback (see e.g., Burrow and Rainone, 2017).

We also analyzed the trait effect of delay discounting, which manifests itself in the association of delay discounting of one outcome with that of other outcomes. The present data provide strong support for a trait influence, as delay discounting of money, followers and likes were all correlated, thus confirming hypothesis H2. This cross-outcome reliability has been shown in many previous studies (Odum et al., 2020) and indicates that state-dependent shifts of delay discounting (caused by e.g., different contexts or rewards) occur at different baselines, which represent the trait influence. Thus, a highly impulsive person with regard to money might behave slightly differently in an Instagram context but will nonetheless be characterized by rather impulsive choices. The correlation between delay discounting of followers and likes was the strongest, which is an intuitive result given that both types of rewards are social in nature and stem from the same platform. Surprisingly, money discounting and follower discounting had the weakest association in this study. The number of Instagram followers associated with an account is a rather stable metric which may be considered an account balance, thus sharing some characteristics with money. The number of likes a user receives, on the other hand, matters most immediately after content was posted since this signals the Instagram algorithm that the post is attractive, resulting in content to be displayed more prominently. Additional followers are typically received much less frequently than additional likes, whose magnitudes also fluctuate considerably more. Thus, it is somewhat puzzling that, vis-a-vis delay discounting of followers, delay discounting of likes had more shared variance with delay discounting of money. Breaking down delay discounting by reward magnitude showed that inter-correlations were not the highest for matched reward sizes (e.g., small money vs. small followers, medium likes vs. medium followers). This may be an indication that the three rewards were not of equal subjective value to participants. Indeed, reward magnitudes in the three delay discounting tasks were not scaled to equivalent (monetary or subjective) value in this study, precluding a direct comparison of discount rates. Any differences in discount rates may simply be due to different value functions of the three rewards rather than due to differences in reward characteristics *per se* (see Chapman (1996) for a detailed discussion). Our data seem to suggest that delay discounting for the three rewards was different [indicating a state effect (Odum et al., 2020)], but future research should investigate this hypothesis using calibrated reward magnitudes.

A surprising result was that self-control, typically a reliable predictor of delay discounting (Duckworth and Kern, 2011), was not related to delay discounting of money, followers and likes. Hence, our findings did not support hypothesis H3. Since the Brief Self-Control Scale is a self-report instrument, a possible explanation could be that participants did not answer as truthfully in this online study as they might have done in a lab-based setting. Similar reasoning may be applied to the null finding with regard to Instagram screen time. Here, we found that people’s self-reported usage duration of the Instagram app was not associated with any of the three measures of delay discounting, which disproves hypothesis H4. Self-reported screen time has been shown to be an adequate indicator of actual screen time but app-based measures (e.g., Screen Time on iOS or Digital Wellbeing on Android, which have been used in related studies) are more accurate and should be used in combination with self-reports when available (Ohme et al., 2021). Additionally, in this present study we only elicited Instagram-related screen time instead of total screen time. Thus, screen time of gaming apps or other social media, which have previously been shown to be correlated with delay discounting (Schulz van Endert and Mohr, 2020), were not included. When accounting for psychological and demographic variables, we found that the average number of likes a person typically receives on one of their posts is predictive of delay discounting of likes. That is, the more likes a person is accustomed to, the more impulsive they are toward this kind of appreciation. The nature of the immediacy-oriented Instagram algorithm for likes described above may help explain this result. A person typically receiving plenty of likes is probably concerned about receiving likes quickly in order to maximize visibility on the platform’s feed. In contrast, users with few likes usually received for their content seem to place less emphasis on increasing prominence and are thus more willing to wait for a higher number of likes, which are, due to the delay, less likely to boost this user’s popularity. This interpretation is supported by the finding that individuals with higher average like count also reported a greater motivation to maximize followers and likes.

In an effort to understand the observed discounting patterns for Instagram rewards better, we also investigated these in relation to participants’ attitudes and preferences. In spite of the majority of participants in this present study stating that their preference for Instagram likes will decrease in the near future, we did not find an association between people’s future preference for likes and their delay discounting of likes. Participants’ views on the objective future worth of likes turned out to be divided and did not provide any clues about delay discounting of likes either. Further, we found no association between participants’ motivation to maximize likes and delay discounting of likes. When looking at the corresponding statements in terms of followers, we did not find any associations between participants’ responses regarding the future preference, future worth or motivation to maximize and their delay discounting of followers. This certainly does not imply that these factors do not play a role in the context of Instagram rewards; rather, the puzzling results may simply be due to the elicitation method, which will be discussed below.

This study has several limitations, which may be addressed by future research. First, the same participants completed three similar delay discounting tasks in one session, which potentially introduced common method bias (Podsakoff et al., 2003). Future studies could create some temporal separation between the measurements or employ tasks with different (e.g., value-calibrated) reward magnitudes. Second, despite being an efficient instrument, the MCQ has some drawbacks. For instance, the smaller, sooner rewards are always available immediately (as opposed to choices where both rewards become available at different points in the future), bearing the risk of overweighting present bias (O’Donoghue and Rabin, 2015). Also, the identification of non-systematic responses is difficult for extreme discounting; always choosing the smaller, immediate reward as well as always choosing the larger, delayed reward are 100 percent consistent responses but may also stem from a lack of attending to the questionnaire. To address these points, future studies may employ computer-based, titrating delay discounting instruments, e.g., by Du et al. (2002), which adjust the reward amounts based on the participant’s previous responses. Third, all rewards were hypothetical in this study. While this has been shown to be unproblematic for monetary rewards, we cannot rule out the possibility that participants would have behaved differently if they had actually received followers and likes for their Instagram account. Since purchasing and awarding followers and likes is commercially available, future studies could replicate our findings with real payoffs. Fourth participants’ attitudes and goals toward Instagram were elicited in a brief and simple manner. To be able to shed more light on the reasons behind discounting of Instagram rewards, more elaborate tools to assess future preference, objective future worth and motivation to maximize rewards seem necessary. Fifth, our delay discounting measurement method did not allow for a direct comparison of discount rates for the three reward types. Non-monetary rewards are typically discounted less steeply than money [i.e., a state influence of delay discounting (Odum et al., 2020)] and the replication of this effect in the context of social media rewards lends itself as a topic for future research. Lastly, only participants who are regularly exposed to Instagram followers and likes, i.e., active users, were included in this study. Based on our data, we cannot make inferences about delay discounting of Instagram rewards by non-users or people unfamiliar with the platform. However, it is unlikely that the latter group would consider Instagram followers and likes as rewards, rendering an analysis meaningless. Instead, future studies could employ other kinds of social media rewards, such as Twitter followers and likes or Facebook likes, as these platforms tend to be used by different population groups.

Our study provided initial evidence that Instagram followers and likes, as novel yet widespread reward types, are processed in a similar way as previously studied rewards in the context of delay discounting. A person’s general, trait-like impulsivity remains recognizable in the discounting patterns of Instagram rewards. Further research is needed to determine if these rewards also cause any temporary shifts in delay discounting on the one hand and to clarify the relationships between delay discounting of Instagram rewards and self-control as well as actual screen time on the other hand.

## Data Availability Statement

The original contributions presented in the study are included in the article/Supplementary Material, further inquiries can be directed to the corresponding author.

## Ethics Statement

The studies involving human participants were reviewed and approved by the German Association for Experimental Economic Research. The patients/participants provided their written informed consent to participate in this study.

## Author Contributions

TS created the study concept, programmed the experiment, collected, and analyzed data, and wrote the manuscript. PM provided study funding, assistance during data analysis, and feedback for the manuscript. Both authors contributed to the article and approved the submitted version.

## Conflict of Interest

The authors declare that the research was conducted in the absence of any commercial or financial relationships that could be construed as a potential conflict of interest.

## Publisher’s Note

All claims expressed in this article are solely those of the authors and do not necessarily represent those of their affiliated organizations, or those of the publisher, the editors and the reviewers. Any product that may be evaluated in this article, or claim that may be made by its manufacturer, is not guaranteed or endorsed by the publisher.
